# Book Review

**Published:** 1906-03

**Authors:** 


### Reviews of Books.

**A Manual of Chemistry, Inorganic and Organic.** By ARTHUR P. LUFF, M.D., and FREDERIC JAMES M. PAGE. Third Edition. Pp. xv., 555. London: Cassell & Co. 1905.

This is one of the worst types of cram books, in which both theory and even bare statements of fact are either incompletely or entirely incorrectly stated. The account of the atomic theory is poor in the extreme, and no student of chemistry could gather from it a sound or even correct knowledge of one of the most fundamental principles of the science. To state, for example, that the fact that all gases obey the laws of Boyle and Charles is a "proof" of Avogadro's hypothesis is entirely incorrect; further than this, to talk about the "proof" of such an "hypothesis" is illogical in the extreme. Although in the preface the authors say that they propose to bring together "those portions of the science that directly or indirectly bear on the study and practice of medicine," yet the problems of solution are not considered worthy of mention; and the ionic theory, which has revolutionised the science, is not even alluded to. On these questions Hamburger has just published his third large volume, showing their great importance in medical learning; this alone is an indication of the rapid growth of this aspect of chemistry, and of its value in medicine. The necessity of familiarising the student with the reasons for assigning formulæ to various gaseous compounds is entire y overlooked, and on the same page the formulæ $CO_2$ and $Si O_2$ are to be found without any explanation of their entirely different meanings. The discussion of the theory of Valency might have been written twenty years ago. Incorrect statements abound: The explanation of the action of aqua regia is incomplete; iodine is not monovalent only; the formulæ for the oxides of sodium and potassium are incorrect; the series of equations representing the formation of sulphuric acid are incomplete. To state that the main function of the nitrogen of the atmosphere is to dilute the oxygen, without also saying that it is the world's reservoir of that substance, is most misleading. When discussing the amorphous forms of carbon, not to allude either to coke or say gas carbon, but to state under a special heading that tinder is a variety, appears childish. The discussion of the important question of isomerism is surprising, the first example given, viz. oil of lemons and oil of turpentine, is amazing, and the subdivision of isomeric bodies is absolutely misleading. The problem of the structure of a gas and candle flame is incompletely given, and the modern views of combustion are not considered worthy of mention. To give the production of chloroform from methane as a "test" for the latter substance is absurd. The method described for determining the melting-point of a substance is amusing, and utterly valueless in the large majority of cases. The positions of the hydroxyl or carboxyl groups in salicylic acid are not given. The statement that aniline on oxidation gives the aniline dyes is so misleading as to be practically incorrect. Uric acid has not been synthesised by "heating glycol with urea." But enough has been mentioned to show the value of this production, which certainly ought not to get into the hands of medical students.

**Golden Rules of Dental Surgery.** By CHAS. W. GLASSINGTON. Pp. 69. Bristol: John Wright & Co. [N.D.].—This little work contains many useful hints which students will do well to follow, and possibly practitioners may with profit recall. The remarks relative to the dentist's manners and general conduct in the surgery, both as to the treatment of his patients and the way he should carry out his work, are very much to the point. The perusal of this little book will remind the reader of the necessity of avoiding the unfortunate habits the writer mentions, and into which it is so easy for all those who are working alone every day to fall.

**Sanitary Inspector's Handbook.** By ALBERT TAYLOR. Fourth Edition. Pp. x., 455. London: H. K. Lewis. 1905.—This book has already taken a place amongst the reliable guides for sanitary inspectors, and has been brought well up to date in the fourth edition. It can be safely recommended as a concise and accurate handbook.

**Guy's Hospital Reports.** Vol. LIX. London: J. & A. Churchill. 1905.—A great part of the present volume (about one-third) is taken up by an article on "Traumatic Subdural Hæmorrhage," by W. H. Bowen, M.S., based on the examination of seventy-two collected cases. It is a very exhaustive article, and has been most carefully prepared. The only other article of importance is on "Growths of the Kidney and Adrenals," by Owen Richards, M.B., B.Ch. In this are collected particulars of all the primary renal and adrenal growths which occur in the records of Guy's Hospital between the years 1826 and 1904. Clinical and *post-mortem* records are full; histological examination is lacking in the earlier cases, and not always decisive in the later ones. The value of the series is chiefly clinical, and as the majority of the cases (seventy) were not operated on, it gives a fair picture of the natural course of the disease. We may perhaps express regret that the present volume contains so little of the work of the staff of Guy's Hospital. Probably their time is now so fully occupied by the drudgery of teaching, that they are less able to indulge in original observations than their distinguished predecessors who made the earlier volumes so famous.

**The Lister Institute of Preventive Medicine.** Collected Papers, No. 1. London. 1904.—The seventeen papers in this volume are full of interest. Dr. George Dean contributes further observations on the Leprosy-like Disease of the Rat, to which special attention was called by Stefansky in 1903 while working at Plague in Odessa; the Mechanism of Agglutination is discussed by Mr. J. A. Craw. Papers by Mr. Plimmer on the Treatment of Cancer with Radium Bromide, and by Dr. C. J. Martin on questions bearing on Antitoxic Immunity, and sundry others on matters of chemical and physiological interest are here reprinted and collected from the various scientific journals.

**Transactions of the American Dermatological Association.** Twenty-eighth Annual Meeting, June, 1904. New York: The Grafton Press.—Though not perhaps so interesting as usual this year, the *Transactions of the American Dermatological Association* repay for the trouble of reading. Always presented in a readable form, the book shows at a glance what are the main problems exercising the minds of our Western colleagues. Of the papers included the following are worthy of notice:—Wende and Bentz on the Pathological Analysis of Five Tumours removed from a case of Rhinophyma; Corlett on Post-vaccinal Eruptions, an attempt at classification according to characteristics and ætiology; Klotz on two undoubted cases of re-infection of cured Syphilitics; and especially two papers by Fordyce and Engman on the Affections of the Mucous Membranes in their relation to skin diseases. There is no doubt but that this subject is one of increasing importance. It is, indeed, a matter of surprise that in the past so little importance has been attached to the visible mucous membranes. This partly, no doubt, is because of the relatively rare appearance of lesions in the mouth, but is also due to the fact that the altered appearance of lesions on mucous membranes has led them to be disregarded or misunderstood. It seems strange that Koplik's spots should not have been earlier described, whilst the appearance of lesions of lichen planus in the mouth presents a difficulty of diagnosis from syphilis or leuko-keratosis which may have led to error. But that the mucous membrane, whether visible or not, frequently shares with or alternates with skin in its involvement in diseased conditions is now recognised. The alternations between eczema and asthma have often been discussed, and the "gastric crises" in angio-neurotic œdema have of late been described by Osler and others. Lupus erythematosus is now recognised on the mucous membranes of the nose and mouth, while psoriasis, seborrhœa, impetigo, pemphigus, xanthoma, lupus vulgaris, and leprosy have their places. In not a few cases it would seem that the mucous membranes serve as the portal of entrance of the disease into the system, which disease subsequently will affect the skin more obviously than the mucous membranes themselves. The classification of the combined returns of the dermatological cases for the year always proves of service.

**Transactions of the American Surgical Association.** Vol. XXII. Philadelphia: William J. Dornan. 1904.—Each year we look forward to the publication of these *Transactions* with a confidence born of past experience that we shall find in them articles dealing with the latest developments in surgery, written by experienced and competent surgeons. The present volume well maintains the high standard set in past editions. All the papers well repay perusal, and some are of very exceptional interest—for example, that upon the Ankylosis of Joints and Arthroplasty, by J. B. Murphy. Among the many other subjects upon which light is thrown we may mention the following:—Duodenal Ulcer, by Christopher Graham and William J. Mayo; Removal of the Shaft of the Tibia for Osteomyelitis, by G. B. Johnston; Cleft Palate, by T. W. Brophy; Surgery of Nerves, by C. A. Powers, and also by Emmet Rixford; Operations for Primary Carcinoma of the Liver, by L. Freeman; and Thyroidectomy for Exophthalmic Goitre, by C. H. Mayo.

**Transactions of the College of Physicians of Philadelphia.** Vol. XXVI. Philadelphia. 1904.—An interesting memoir of Robert Bartholow, M.D., commences the volume. From it we are gratified to note that "the best part of his work has found a place in the sum of knowledge available for curing disease, and that the inspiration of his teaching still lives in the precepts of his books and in the practice of thousands of his students." What better proof can there be that he left the world a little better than he found it! One of the most important papers is that by Drs. Edsall and Ghriskey on "A Small Hospital Epidemic of Pneumococcus Infection, with Remarks on the Transmission of Pneumonia and the Methods by which it can be Prevented." It is commonly admitted that pneumonia does not often appear in epidemic form, and that contagion can only rarely be traced; but nevertheless in recent years it has been abundantly shown that pneumonia is an infective disease, and that certain well-recognised bacteria are its usual causes. The literature of the subject leaves no room for doubt that infection may occur (1) through widespread atmospheric dissemination, (2) through the agency of some local source, and (3) through direct contagion from persons sick of the disease. Notwithstanding these admitted facts, "the attitude of the profession toward the control of pneumonia has been somewhat passive; for, in spite of this general knowledge, there are certainly few physicians who adopt more active precautions to prevent the spread of the disease than to have the sputum from pneumonia patients destroyed, and perhaps to utter a gentle warning as to the possible danger of very close contact with those sick of the disease." Accordingly it seems necessary that the profession should be urged to deal more decisively with this important subject, and that our only methods of controlling infections by means of the use of the general principles of preventive medicine should be enforced. Much has already been done as regards tuberculosis, but the fact that these measures may be less successful in pneumonia than in tuberculosis is no argument against their adoption if they are likely to meet with any success at all.

**The Edinburgh Medical Journal.** New Series. Vol. XVII. Edinburgh and London: Young J. Pentland. 1905.—This, the centenary volume, opens with an introductory note by the editors, recalling the most important historical events during the hundred years of the life of the *Journal*. It is illustrated with numerous portraits of former editors and the first publisher.

**E. Merck's Annual Report.** Vol. XVIII., 1904. Darmstadt. 1905.—This report, on the advancements of Pharmaceutical Chemistry and Therapeutics, is manifestly an outcome of an honest desire to furnish a brief and impartial review of the therapeutic acquisitions of the last twelve months. The report is widely appreciated and much valued as a book of reference, inasmuch as it gives the latest views on the numerous new drugs which crowd upon us with claims which are often misleading and not verified by increased experience. A true spirit of impartiality pervades the book. Not only are the new products of the Merck firm described fully, but interesting or valuable preparations from other sources are also described, and the older every-day preparations are included whenever their therapeutic use presents fresh aspects.

**Golden Rules of Medical Practice.** By Lewis Smith, M.D. Sixth Edition. Pp. 126. Bristol: John Wright & Co. 1905.—The author remarks in his preface that "amid so much that is theory, the facts and necessary rules of the practice of medicine tend to become obscured." The practical rules, which the author gives in 126 small pages, are founded on much experience as a practitioner and teacher; they are for the most part elementary axioms intended to assist those who like concentrated pabulum, and who will not give themselves the trouble to read and digest the ordinary text-book.

**The Modern Nursing of Consumption.** By Dr. Jane Walker. Pp. 47. London: The Scientific Press Limited.—The nurse who takes pains to make herself acquainted with the elementary facts of medicine and surgery, in so far as they bear on the nursing of patients coming under her care, nearly always becomes much more helpful to the practitioner as well as to the patient, and, with chronic complaints such as consumption, a nurse who, in addition to a sound, general training, has acquired some special experience of the nursing of consumptives, may be of very great assistance. Dr. Walker has had much experience in the treatment of these cases, and her long acquaintance with the need for specially training nurses for patients undergoing open-air methods has enabled her to produce a small manual of very great practical value.

**A Junior Course of Practical Zoology.** By the late A. Milnes Marshall, M.D., D.Sc., and the late C. Herbert Hurst. Sixth Edition. Revised by F. W. Gamble, D.Sc. Pp. xxxiv., 490. London: Smith, Elder & Co. 1905.—In the case of a new edition of a book so well known and universally used as "Marshall and Hurst" a review is unnecessary, all that is required is to indicate in what respects the new edition differs from the previous one. In the present edition, which is edited by Dr. F. W. Gamble, the most noteworthy feature is the addition of accounts of *Monocystis*, *Coccidium*, and *Obelia*—types which have been introduced into the revised Preliminary Scientific course. These accounts are illustrated by two good figures. *Nereis*, whose external characters are now also included in the Preliminary Scientific course, is not described. The additions referred to have not increased the bulk of the book, as certain portions are now printed in smaller type than they were in earlier editions.

**The Biology of the Micro-organism of Actinomycosis.** By JAMES HOMER WRIGHT, M.D. Pp. 56. 51 plates. Publication Office of the *Journal of Medical Research*, Boston, Mass. 1905. —This monograph is No. 1 of Vol. I. of the publications of the Massachusetts General Hospital, and is a careful and elaborate contribution to a confessedly difficult subject. Possibly the study of bacteriology has not advanced sufficiently far for any complete system of classification, at any rate authorities are still at variance on many important points. There are, no doubt, numerous related organisms in the genus commonly known as actinomyces; the main contention of the author of this monograph is that only one of these, and that not the one usually so accredited, is the pathogenic agent in the disease of actinomycosis, bovine and human. The main points of differentiation, according to Dr. Wright, are the appearance of characteristic granules (*drusen*) in the lesions, and the cultural peculiarities of the organism in a pure state. After describing with commendable detail his own experiments and results, he reviews the literature with the object of showing that a large number of observers have isolated an organism more closely resembling the one he describes than that described by Bostroem, Hartz, and Gasparini, which has been regarded as the true actinomyces bovis. The organism described by the latter he regards as a cultural contamination. Considerable difficulty exists in inoculating experimental animals, which adds to the uncertainty which surrounds the subject. In addition to purely descriptive and experimental results, Dr. Wright advances a theory, at present only conjectural, that the fungus is normally saprophytic in the mouth and digestive tract, and does not grow outside the animal body. He regards the barley-corn so frequently associated with the lesions as a foreign body acting as a predisposing irritant, and not as a true carrier of the organism. He also proposes the name nocardia (which is already used by some authors) instead of actinomyces to designate the genus. The Massachusetts General Hospital is to be congratulated in being able to inaugurate their publications with so scholarly and scientific a contribution to a difficult and important subject.

**Indigestion.** By GEORGE HERSCHELL, M.D. Third Edition. Pp. 293. London: Henry J. Glaisher. 1905.—Owing to recent advances in this field, it has been found needful to practically rewrite the whole book, and its usefulness has been increased by the addition of an appendix of 160 pages on the preparation of food by cooking, with especial reference to its use in the treatment of affections of the stomach. Chapters on Gastric Myasthenia and Neurasthenia are followed by one on the Modern Intragastric Methods of Diagnosis. This we consider to be the most valuable part of the book, inasmuch as it gives practical and reliable information on methods which are as yet in little use, and of the value of which there is still much doubt and misconception. The author lays down the dictum that "in any obscure case of gastric disease we require to know the following points before we can make a diagnosis: (1) The position of the stomach and its apparent size; (2) the degree of muscular sufficiency of the stomach; (3) the condition of the gastric juice as to quantity and quality; (4) the presence or absence of fermentation in the stomach; (5) the presence in the stomach of mucus in excess, and in certain cases also blood, pus, and the Oppler-Boas bacillus and fragments of mucous membrane." We are surprised to find that the whole subject of pre-digestion of food, and the use of the various digestive ferments, either for the preparation of food or to assist its digestion within the body, are scarcely mentioned. Benger's pancreatised lentil flour is mentioned on pages 242 and 243, but we do not find any reference otherwise to the dietetic or medicinal uses either of the pancreatic or gastric products. There is no mention even of peptonised milk in either the index of the book or the appendix. This is an omission which a future edition may shortly supply; meanwhile we are informed that gruels and purées may be plain or peptonised, but no information as to the process of peptonisation is afforded.

**A Manual of Surgery.** By William Rose, M.B., B.S., and Albert Carless, M.S. Sixth Edition. Pp. xiv., 1350. London: Baillière, Tindall, & Cox. 1905.—Although containing nearly 200 pages more than the first edition, the present issue is actually smaller in size (except for the width of the page), a result which has been achieved by using a thinner paper and by including many of the pictures in the text. Professor Carless is responsible for this new edition, which thoroughly deserves the praise which we have before bestowed on the book. Dr. W. D'Este Emery has supervised the pathological portions, and has rewritten or added chapters dealing with bacteriology and the blood. The sections on modern surgical technique and the surgical aspects of gynecology are welcome additions to a book which can be thoroughly recommended as a reliable, readable, and comprehensive text-book.

**A Plea for the more Energetic Treatment of the Insane.** By Charles Williams. Pp. 51. London: Henry J. Glaisher.

1905. The title of this brochure appeals to all those who are engaged in the treatment of the insane, and the author has made an earnest and whole-hearted effort to encourage them to increase the recovery rate by perseverance, especially in the less commonly used methods of treatment, and for the attainment of this object he must not be blamed for showing a certain amount of justifiable optimism. There can be no question of the value of treatment by thyroid extract, electricity, the prolonged warm bath, and the Turkish bath in certain selected cases, but the author especially urges their use in certain cases which are generally considered less suitable. No doubt better results would be obtained if the difficulties of carrying out the treatment could be more easily surmounted. In the treatment of insanity by counter-irritation, or by inducing in the body some somatic disease, the question is more difficult. Unfortunately it is the diseases which are serious to life which produce the most beneficial effects, and there must always be great hesitancy in inducing them artificially owing to the lower vitality of the insane and the greater difficulty in nursing, and the author admits this treatment is hardly ripe for wholesale trial. As regards "treatment by persistent argument, coupled with practical proof of the falsity of any delusion," the author states that it is "valuable in thousands of so-called insane people in various asylums, who, except on the subject of their delusions, are sensible and sane enough." In our opinion it is almost valueless except in earliest stages—before certification.

**Electrical Methods in the Treatment of Affections of the Stomach and Intestines.** By GEORGE HERSCHELL, M.D. Pp. 108. London: A. Siegle. 1905.—This little volume is a reprint of a series of papers published in the *Journal of Medical Electrology and Radiology*, to which has been added a series of clinical histories illustrative of the value of electrical treatment in the various disorders of the gastro-intestinal tract. The papers here brought together are, according to the author, "not intended for the expert, but are written for the now rapidly-increasing number of medical men who have become interested in the subject and, appreciating the value of electricity, wish to make use of it in their daily work." It is interesting to find that the author thinks that with the galvanic, the Faradic, the galvano-Faradic, and sinusoidal currents we can do as much for our patients as we can with the electrical forms of energy of high potential and frequency, and, as he points out, these latter are much more dangerous in unskilled hands. Judging from the clinical records at the end of the book, electrical methods in properly-skilled hands are a very potent means of cure in chronic disorders of the digestion, and the publication of these papers in book-form will do useful service in drawing attention to a somewhat neglected method of treating this troublesome class of cases.

